# A pH-Induced Reversible Assembly System with Resveratrol-Controllable Loading and Release for Enhanced Tumor-Targeting Chemotherapy

**DOI:** 10.1186/s11671-019-3139-z

**Published:** 2019-09-06

**Authors:** Qingkai Zheng, Wenjing Cheng, Xiaoping Zhang, Runxia Shao, Zhongdong Li

**Affiliations:** 1 Department of Respiratory Medicine, Jiaozuo People’s Hospital, Jiaozuo, Henan 454000 China; 2 Health Management Center, Jiaozuo People’s Hospital, Jiaozuo, Henan 454000 China; 3 Department of Respiratory Medicine, The Second Affiliated Hospital of Zhengzhou University, Zhengzhou, Henan 450000 China; 4 Department of Hematopathology, Jiaozuo People’s Hospital, Jiaozuo, Henan 454000 China

**Keywords:** Resveratrol, Ferritin, pH-sensitivity, Apoptosis, Nanomedicine

## Abstract

In this report, we present a pH-induced reversible assembly system (PIRAS) based on ferritin (Ft) for targeted tumor therapy. It has been developed to easily load and release of the anticancer drug resveratrol (RV), based on its natural pH-sensitivity and unique hollow cavity of Ft. A tumor-specific target peptide Arg-Gly-Asp (RGD) was conjugated onto the surface of RV-loaded Ft (RV@Ft), to form biocompatible nanoparticles (RV@Ft-RGD). The pH-sensitivity of Ft allows it to be denatured into a hollow porous nanosphere under acidic condition and renatured into a sealed hollow nanosphere under neutral condition. Using pH manipulation, RV@Ft-RGD, with a ~ 21 nm diameter, showed a high RV loading ratio of 79.6%. pH-triggered RV release was then measured at a ratio of 50.3% at pH5.0 over 24 h. Under neutral condition, the RV@Ft-RGD showed excellent stability over 20 days. Confocal fluorescence imaging showed that RV@Ft-RGD had a high cell uptake ratio and co-localization with the lysosome, mainly due to the RGD-mediated target effect. Based on the high drug loading, pH-triggered release, and tumor cell targeting effect, RV@Ft-RGD showed great cell-killing ability in vitro and in vivo. The biocompatibility in vitro and in vivo was also demonstrated to be excellent, without systematic toxicity. The design concept of PIRAS based on Ft significantly inhibits tumor growth and simultaneously further broadens the application of Ft in nanomedicine.

**Electronic supplementary material:**

The online version of this article (10.1186/s11671-019-3139-z) contains supplementary material, which is available to authorized users.

## Introduction

Lung cancer is one of the deadliest solid malignancies in humans. With the increased deterioration of the environment and other factors, the incidence of lung cancer has increased year by year, and its 5-year survival rate is only 17.4% [1, 2]. At present, although traditional clinical treatments such as surgical resection, radiation therapy, and standard first-line chemotherapy are available, the median overall survival rate of lung cancer patients still needs to be improved [3, 4]. Therefore, more effective and safer treatments urgently need to be developed to cure this deadly disease. Currently, although low target-effects and high side-effects of many chemotherapeutic drugs have limited their utility in chemotherapy [5, 6], new chemotherapeutic agents are constantly being developed, especially in the emerging field of nano-drugs [7–10]. Resveratrol (RV) is an extract of natural plants such as grapes and soybeans, which has been widely used in clinical settings to promote platelet aggregation, inhibition of vasodilation, reduction blood viscosity, and the like [11–15]. In recent years, it has also been found to have a strong anticancer effect [16–18]. However, as a potential small molecule drug, RV also has some disadvantages as other first-line chemotherapeutic drugs—such as poor solubility, short half-life of blood circulation, and lack of tumor selectivity [19, 20]. RV-based nano-drugs have been developed in recent years to overcome these shortcomings and to enhance its therapeutic effects [21]. Various kinds of nanocarriers have been used to load RV, including liposomes, serum albumin, carbon materials, two-dimensional transition metal dichalcogenides (2D-TMDs), and others [21–25]. Although these nanocarriers have been reported to efficiently enhance the therapeutic effects of RV, they still show some deficiencies—such as high-efficiency active trigger-release capacity for liposomes and serum albumin, and potential long-term systemic toxicity for carbon materials and 2D-TMDs [26, 27]. Therefore, there is still a demand for more qualified nanocarriers.

Ferritin is a natural nanocage protein with a lumen of approximately 8 nm, formed through the interaction and assembly of 24 subunits of heavy and light chains [28, 29]. Because it is an endogenous protein, ferritin has excellent biocompatibility and safety. In addition, there is a report that ferritin is a naturally pH-sensitive protein that can be reversibly denatured and reassembled as its environment changes from acidic to alkaline condition [30]. When the pH was acid, the disassembled rod-like oligomers recovered only to the headset-shaped structure, and the disassembled headset-shaped intermediates recovered only to the hollow spherical structure [28–30]. This unique pH-triggered assembly and disassembly behavior makes ferritin an ideal drug delivery system. Recently, Zhang et al. reported that doxorubicin (DOX) molecules can be encapsulated in ferritin and successfully released by pH regulation for tumor therapy [28].

In this study, we aimed to develop a ferritin-based pH-induced reversible assembly system (PIRAS) to control RV loading and release for enhanced tumor-targeting chemotherapy. The surface of the ferritin sphere was linked with the tumor-targeting peptide RGD. The resultant nano-drug RV@Ft-RGD was demonstrated to be stable in neutral and alkaline environments (pH > 7.4), and only release RV in acidic environments (pH < 7.4). When further characterized, RV@Ft-RGD showed the following benefits in vitro and in vivo: (1) RV@Ft-RGD targeted tumor cells and accumulated in the lysosome (acidic environment), where facilitated the release of more RV into the cytoplasm; (2) the ferritin carrier significantly enhanced the blood half-time of free RV, improving drug retention in the systemic circulation to facilitate the accumulation of drugs in tumor sites; (3) architectural stability of the ferritin prevented RV burst leakage during the delivery process; (4) RV@Ft-RGD showed great in vitro and in vivo biocompatibility. Therefore, owing to these merits, RV@Ft-RGD displayed splendid antitumor therapeutic properties, which show great potential for future clinic translations.

## Materials and Methods

### Materials

Ferritin, resveratrol (RV, ≥ 99%) were from Sigma-Aldrich. 1-ethyl-3-(3-dimethylaminopropyl) carbodiimide (EDC), N-hydroxysuccinimide (NHS), and fluorescein isothiocyanate (FITC) were purchased from Aladdin Bio-Chem Technology Co., LTD (Shanghai, China). NH_2_-PEG_2000_-RGD was purchased from Hunan Huateng Pharmaceutical Co., Ltd (Hunan, China). DMEM cell media, fetal bovine serum (FBS), and phosphate-buffered saline (PBS) were obtained from Invitrogen (Carlsbad, CA, USA). Cell counting kit-8 (CCK-8) was supplied by Dojindo Laboratories (Japan).

### Synthesis of RV@Ft-RGD

The RV loading was prepared as described according to the previous report [21, 28] with modifications. Firstly, NH_2_-PEG_2000_-RGD (10 mg) was dispersed into the Ft solution (1.5 mg/mL) under the presence of 20 μL EDC (5 mg/mL) and 20 μL NHS (20 mg/mL). The mixture was reacted for 2 h at 4 °С with slightly stirring, and purified by dialysis against distilled water (MW cut off = 5 kDa) overnight, resulting in Ft-RGD nanoparticles. And then, water-insoluble RV was dissolved in DMSO to be 2 mg/mL and was added into the Ft-RGD solution with a final concentration of 1.5 mg/mL. The pH of the mixture was adjusted into pH = 5 to disassemble the polypeptide subunits. Afterward, the pH of the mixture was slowly adjusted to 7.4 with sodium hydroxide (1 M) to resemble the polypeptide subunits. The resulting solution was dialyzed in distilled water overnight to remove free RV molecules to be the end product (RV@Ft-RGD). The amount of loaded RV was detected by UV-vis spectrophotometer (UV3100, Shimadzu, Japan) by monitoring the absorption peak at 306 nm. RV loading ratio was (*A*_a_ − *A*_b_)/*A*_c_, where *A*_a_, *A*_b_, and *A*_c_ represent the weight of the initial, unloaded RV and Ft-RGD, respectively.

### Characterizations

Dynamic light scattering (DLS) method (BI-9000AT, Brookhaven, USA) was used to detect the size and zeta potential of the nanoparticles. The morphology of the nanoparticles was observed by a transmission electron microscope (TEM, JEM-100S, JEOL, Japan). Fluorescence spectra were detected by a Perkin-Elmer LS50B luminescence spectrophotometer. Cellular fluorescence signal was recorded by the commercial laser scanning microscope (LSM 510, Zeiss, Germany) and Flow cytometry (FCM, EPICS XL, Beckman, USA).

### RV Release Study

Five hundred microliters of RV@Ft-RGD solution was placed in a D-tube (MWCO 6–8 kDa, Novagen), and the solution was adjusted to different pH values via different buffers. The solution was then dialyzed at 37 ^o^C. After a different incubation time, 1 mL aliquots of dialysate were removed and replaced with 1 mL of fresh medium. The RV released at different incubation times was determined by a UV-vis spectrophotometer. In addition, RV@Ft-RGD was dissolved in a flushing solution having different pH conditions, including pH 5, 6.5, 7.4, and 8.5. After incubation at 37 °C for 24 h, the size of the nanoparticles was characterized by DLS.

### Cell Culture

The human lung cancer cells A549 and NCI-H358 were obtained from Cell Collection of Chinese Academy of Sciences (Shanghai, China), and cultured in DMEM medium containing 10% fetal bovine serum (FBS) and 1% penicillin streptomycin (PS) in a humidified atmosphere containing 5% CO_2_ at 37 ^o^C.

### Cellular Uptake and Localization of RV@Ft-RGD

As a common used method, FITC was applied to label the nanoparticles. FITC was dissolved in ethanol solution (2.0 mg/mL) and mixed with RV@Ft and RV@Ft-RGD aqueous solution (1.0 mg/mL) under 4-h stir in dark environment at room temperature. The mixture was dialyzed in distilled water overnight to remove the redundant FITC and ethanol, resulting in FITC-labeled RV@Ft and RV@Ft-RGD solution. To confirm the organelle localization of nanoparticles in vitro, the cells treated with FITC-labeled RV@Ft and RV@Ft-RGD for 5 h and stained by lysosome specific dye Lyso Tracker Red (Invitrogen). Afterward, the cellular internalization of RV@Ft and RV@Ft-RGD was observed using by a CLSM. In brief, A549 cells were incubated with FITC-labeled RV@Ft and RV@Ft-RGD (with same concentration of FITC) for 5 h. And then, the cells were treated with Lyso Tracker Red solutions (100 nM) at 37 °C for 30 min. After washing by PBS for three times, the cells were observed by a commercial laser scanning microscope. ImageJ software was used to analyze the fluorescence intensity of cells.

### In Vitro Tumor Chemotherapy and Apoptosis Study

First of all, the cytotoxicity of the carrier Ft-RGD was evaluated by a standard CCK-8 assay (Bestbio, China). A549 and NCI-H358 cells (1 × 10^5^ cells/mL, 0.5 mL) were seeded in 96-well plate and cultured for 24 h. After discarding the old media, fresh media containing 0, 0.01, 0.1, 0.5, and 1 mg/mL of Ft-RGD were incubated with A549 and NCI-H358 cells for 24 h. The cells were gently washed three times with PBS. One hundred microliters of CCK-8 working solution (10% CCK-8 + 90% DMEM) was then added to each well and incubated for 30 min at 37 °C. Absorbance values at 450 nm were measured using a microplate spectrophotometer (Multiskan FC, Thermo Scientific). Photographs of each group of cells were observed with an optical microscope (Olympus).

Various concentrations of free RV, RV@Ft, RV@Ft-RGD, and RV@Ft-RGD + RGD (0, 10, 20, 30, and 40 μg/mL RV equivalents) were treated with A549 cells. After 24 h incubation, the viability of treated cells was analyzed by CCK-8 assay. Meanwhile, these treated cells were dual-stained by apoptosis detection kit (Annexin VFITC/PI) and analyzed by FCM.

### Circulation Time of RV@Ft-RGD in Blood

Free RV or RV@Ft-RGD (6 mg/kg RV equivalent) was injected intravenously into healthy Balb/c nude mice (*n* = 5 per group). After the injection, the venous blood of the mice was collected at different time points and placed in a blood collection tube containing heparin, and then the plasma was separated. The RV concentration in plasma was extracted by acidified isopropanol reagent, and then its absorbance at 306 nm excitation was measured to determine the concentration of RV.

### *Animal Model and In Vivo* Tumor Chemotherapy

Balb/c mice (4–6 weeks old) used in this work were purchased from Charles River Laboratories (Beijing, China). All operations involving animal experiments are strictly in accordance with the guidelines for the care and use of laboratory animals. The guideline was approved by the Animal Care and Use Committee of Zhengzhou University. To establish a subcutaneous tumor model of A549, 2 × 10^6^ A549 cells were injected subcutaneously into the back of the mice. They were then placed in the animal house for 7 to 9 days. The tumor volume calculation formula is length × width^2^/2.

Mice-bearing A459 tumors were randomly divided into five groups. Each group consisted of five mice. The specific groupings are control (saline), RV, RV @Ft, and RV@Ft-RGD. At the beginning of the treatment, the sample was injected once a day through tail vein for three consecutive days. Tumor volume and mouse body weight were recorded every 5 days. After 45 days of treatment, tumors of each group were collected. Tumor tissues were fixed in 10% formalin solution, sectioned, and then subjected to hematoxylin and eosin (H&E) staining.

### In Vivo Biocompatibility

Two hundred microliters of RV@Ft-RGD was injected into healthy Balb/c mice via tail vein (RV dose was 15 mg/kg). Healthy mice injected with physiological saline in the same manner were used as a blank control group. On days 0, 10, and 45 after injection, mouse blood was collected for evaluation of cell counts including white blood cells (WBC), red blood cells (RBC), hemoglobin (HGB), and mean platelet volume (MPV). , mean red blood cell hemoglobin (MCH), hematocrit (HCT), mean red blood cell hemoglobin concentration (MCHC), mean red blood cell volume (MCV), and platelet (PLT). In addition, heart, liver, spleen, lung, and kidney tissues of each group were collected for H&E staining on the 45th day.

### Statistical Analysis

Data were presented as mean ± standard deviation (s.d.). Statistical analysis of the samples was performed using Student’s *t* test, and *p* < 0.05 was considered statistically significant.

## Results and Discussion

### Synthesis and Characterizations of RV@Ft-RGD

**Fig. 1:**
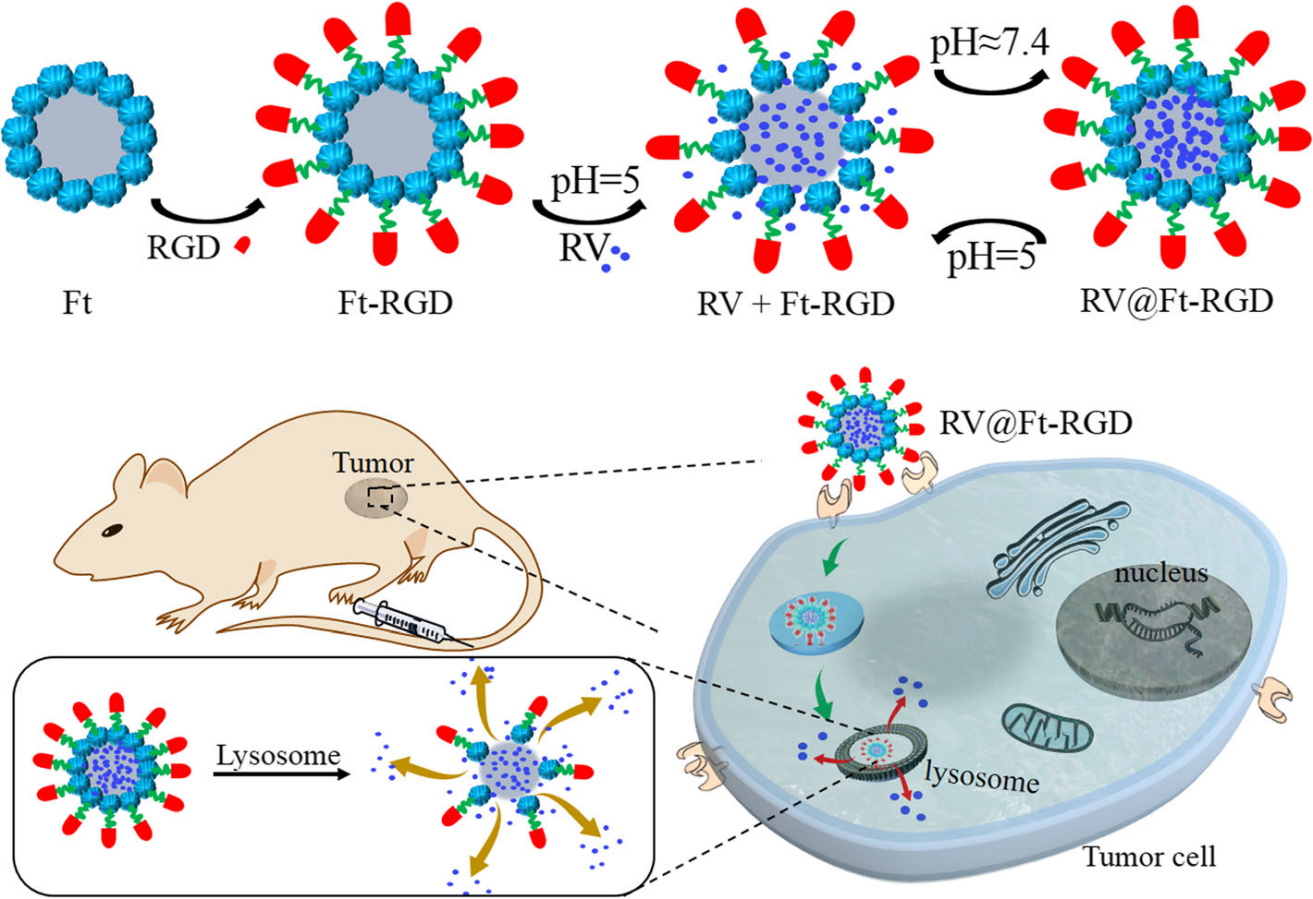
A schematic illustration of RV@Ft-RGD preparation and tumor-targeted chemotherapy

**Fig. 2:**
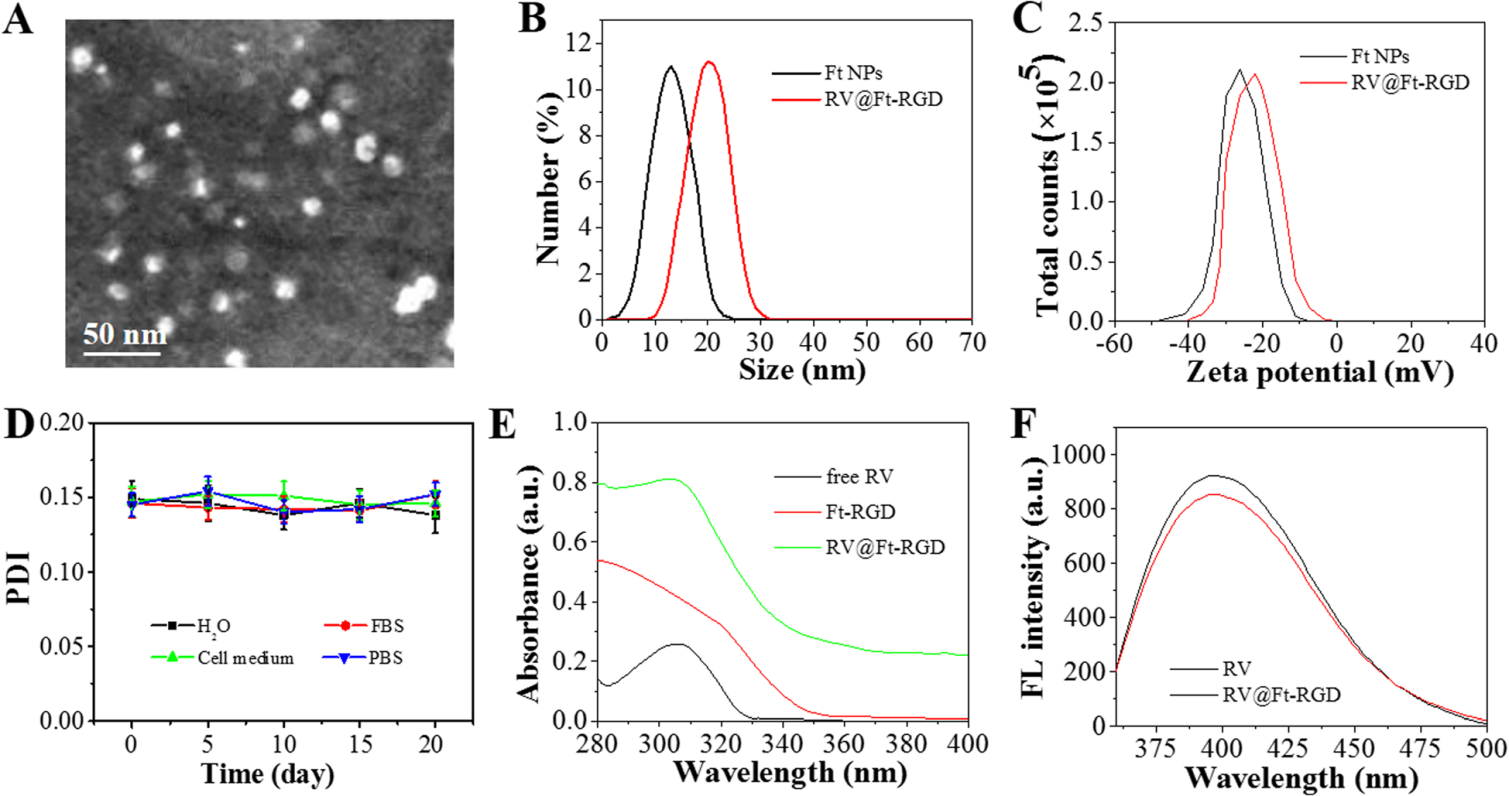
**a** The TEM image of RV@Ft-RGD. **b**, **c** The size distribution and zeta potential distribution of Ft-RGD and RV@Ft-RGD. **d** The PDI change of the RV@Ft-RGD in water, FBS, cell medium, and PBS over 20 days. **e** The absorbance spectra of free RV, Ft-RGD, and RV@Ft-RGD. **f** The absorbance spectra of free RV and RV@Ft-RGD

RV loading and release from RV@Ft-RGD was carried out via the pH-induced reversible disassembly and reassembly of Ft (Fig. 1). Firstly, ferritin was conjugated with the tumor-targeting peptide RGD to form Ft-RGD. This was then redissolved in an acetate buffer (pH = 5) to disassemble the polypeptide subunits and form a hollow pored sphere. RV molecules were then added and allowed to enter into the cavity, after which the solution was slowly adjusted to pH ≈ 7.4 to reassemble Ft, trapping the RV molecules inside the sealed Ft cavity. When the pH of the buffer was then decreased to ~ 5, the nanoparticle pores opened reversibly and released their RV molecules. Figure 2a and Additional file 1: Figure S1 show the SEM and TEM images of the assembled RV@Ft-RGD, which displayed a spherical structure. According to DLS analysis, RV@Ft-RGD particles had larger diameters (~ 22.5 nm) compared to raw Ft nanoparticles (~ 11.8 nm) (Fig. 2b) and had similar zeta potentials as raw Ft nanoparticles (− 29.6 mV) (Fig. 2c). After 20 days, the polydispersity index (PDI) of RV@Ft-RGD in water, FBS, cell media, and PBS showed no significant change (Fig. 2d), indicating that RV@Ft-RGD had remarkable colloidal stability. Figure 2e shows the absorbance spectra of free RV, Ft-RGD, and RV@Ft-RGD. As can be seen, compared with that of RV and Ft-RGD, the absorption spectrum of RV@Ft-RGD exhibited a new peak at 306 nm (originating from RV), indicating the successful loading of RV. In addition, RV@Ft-RGD showed a similar significant emission fluorescence as free RV at 400 nm, when excited by a 325 nm laser (Fig. 2f).

### Drug Loading and Release

**Fig. 3:**
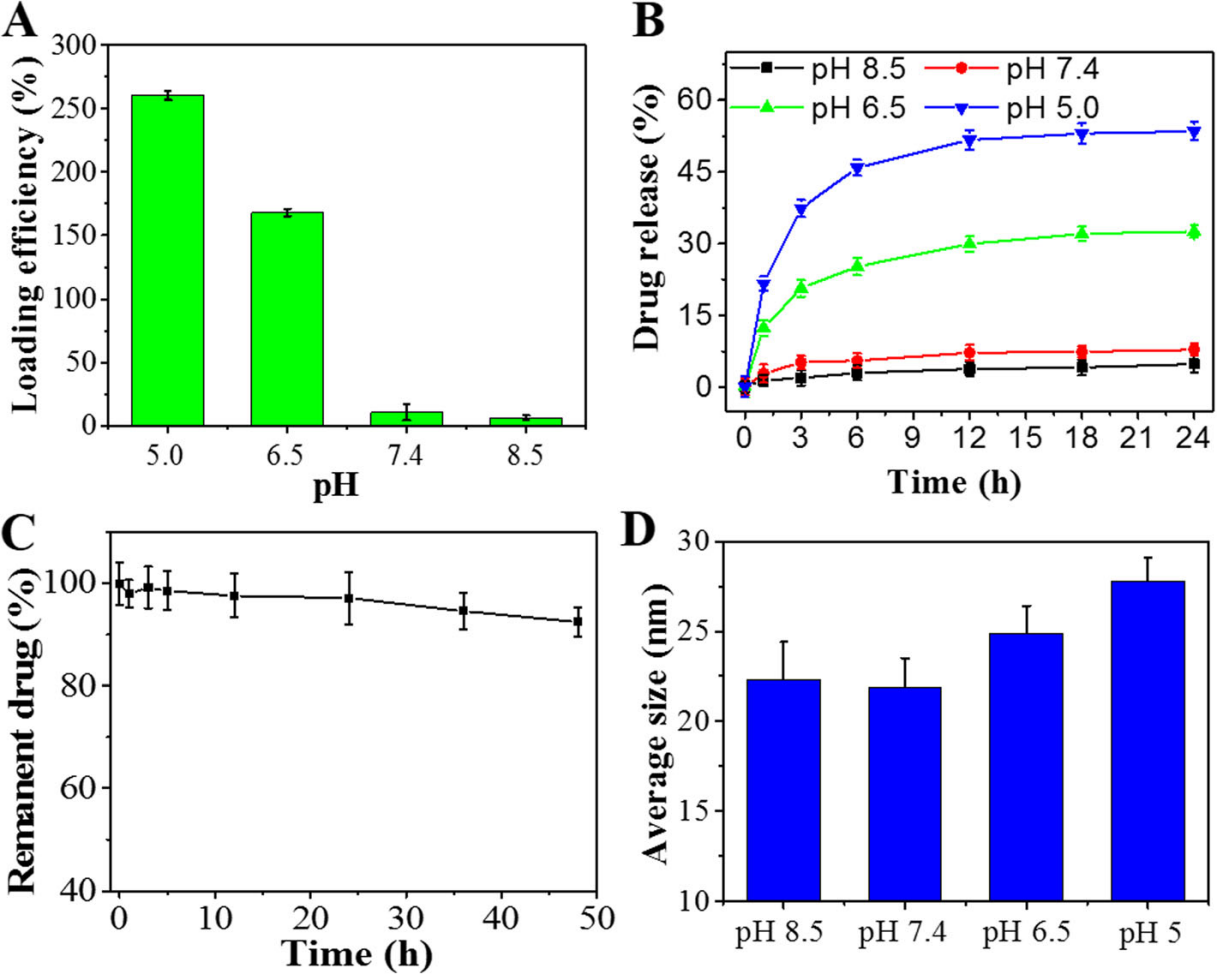
**a** Loading efficiency of Ft-RGD in various pH conditions. **b** RV release profile from RV@Ft-RGD in various pH conditions from 5.0 to 8.5. **c** The permanent RV in RV@Ft-RGD in physiological condition. **d** The average size change of RV@Ft-RGD in various pH conditions from 5.0 to 8.5

The maximum loading capacity of Ft-RGD was found to be 252.6%, likely due to the large cavity inside the hollow Ft-RGD. As Ft is pH-sensitive, we were able to characterize the RV loading capacity under different pH conditions. As shown in Fig. 3a, with the increase of pH from 5.0 to 8.5, RV loading efficiency decreased dramatically. RV release from the complex was also characterized under different pH values from 5.0 to 8.5. A pH value- and time-dependent RV release profile was constructed from this data and is shown in Fig. 3b. The maximum drug release ratio (51.6%) happened in pH = 5.0 for 24 h, which was higher than that in pH = 6.5, 7.4, and 8.5. According to related reports, pH values of 5.0, 6.5, and 7.4 are those typically found in cell lysosomes, tumor tissues, blood, and the normal physiological environment, respectively [31]. Under physiological condition (pH7.4), the RV drug content of RV@Ft-RGD remained high even after 50 h (Fig. 3c), suggesting that the RV in RV@Ft-RGD is stable and does not leak out easily. In addition, we investigated the change in diameter of RV @ Ft-RGD under several different pH conditions. After incubation at 37 °C for 12 h, DLS analysis showed that the diameter of RV@ Ft-RGD was almost constant, at around 23 nm at pH8.5 and 7.4. When the pH value was dropped to 6.5 and 5.0, the diameter of RV @ Ft-RGD increased to 25 nm and 28.6nm respectively (Fig. 3d). These results confirm the behavior of pH-induced reversible disassembly and reassembly of RV@Ft-RGD, which is beneficial to controllable drug loading in vitro and drug release in tumor tissue.

### In Vitro Cellular Uptake and Localization

**Fig. 4:**
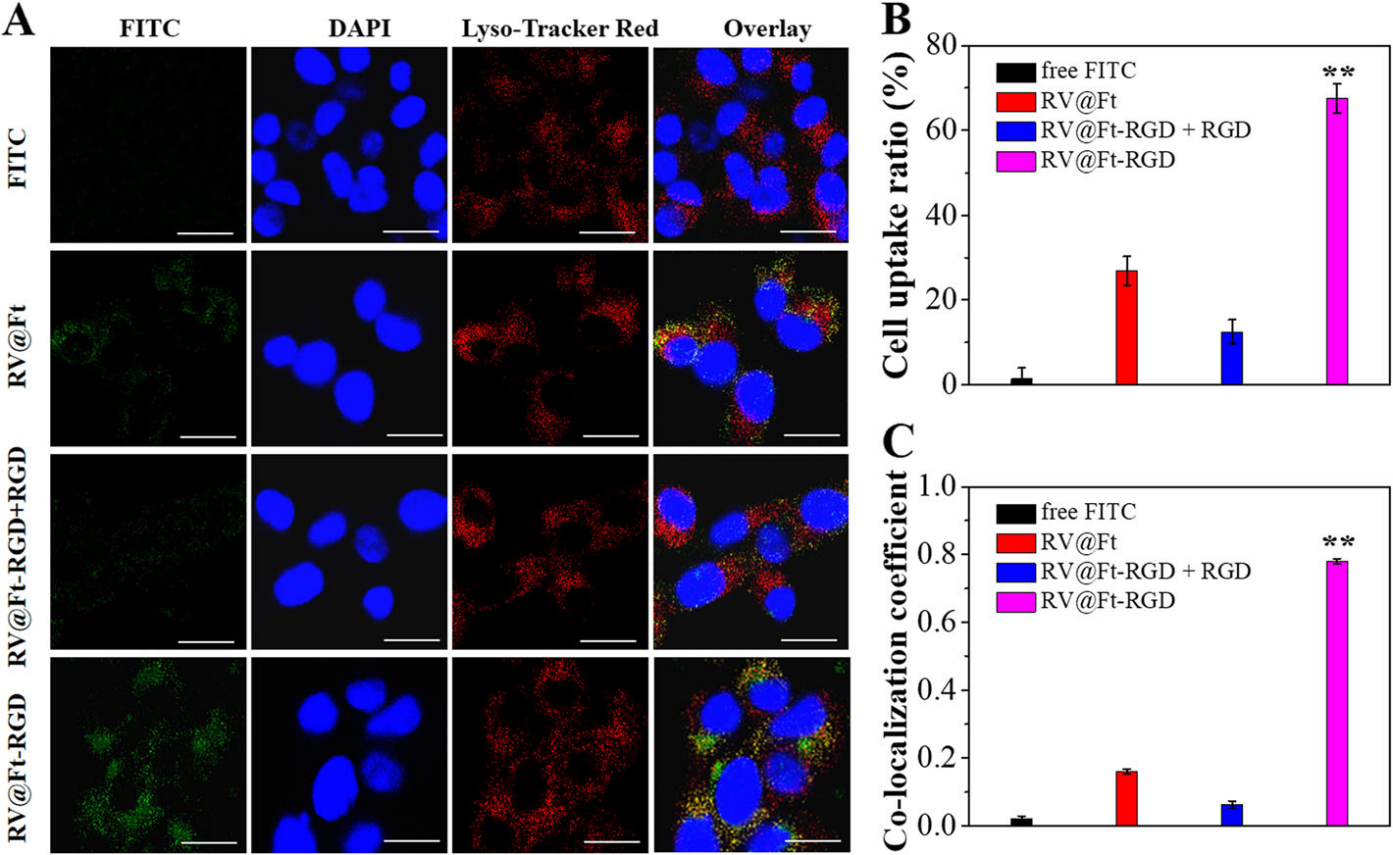
**a** Fluorescence images of A549 cells after incubation with free FITC and FITC labeled RV@Ft, RV@Ft-RGD + RGD, and RV@Ft-RGD, respectively. Scale bar = 60 μm. **b** FCM measurement of cellular FITC fluorescence intensities in A549 cells after incubation with free FITC and FITC labeled RV@Ft, RV@Ft-RGD + RGD, and RV@Ft-RGD, respectively. **c** Co-localization coefficient of cellular fluorescence of FITC and Lyso Tracker Red. **P < 0.01, compared with other groups, respectively

In vitro cellular uptake and localization of RV@Ft-RGD was then evaluated. Firstly, RV@Ft and RV@Ft-RGD were labeled by FITC via hydrogen bond interaction and physical absorption. As shown in Fig. 4a, free FITC-treated cells showed negligible cytoplasmic fluorescence signals. RV@Ft-RGD-treated cells, by contrast, displayed intense FITC green fluorescence, which was higher than that of RV@Ft-treated cells. Notably, RV@Ft-RGD-treated and RGD pre-treated cells both showed low green fluorescence. From these results, we can conclude that RV@Ft-RGD was taken up by cells in high quantities, likely through a RGD-mediated active target effect. These treated cells were also analyzed by FCM, the results of which are shown in Fig. 4b. Cellular fluorescence intensity statistical results suggest a similar conclusion.

To study the organelle localization of RV@Ft-RGD, a lysosome-specific staining dye (Lyso Tracker Red) was used to stain the cells incubated with the nanoparticles. As can be seen in Fig. 4a, the cytoplasm showed strong red fluorescence in all groups. After merging with FITC green fluorescence, RV@Ft-RGD showed a most intense yellow (green + red) fluorescence in the cytoplasm among these groups. Based on statistical analysis, the RV@Ft-RGD-treated group had the highest co-localization coefficient between FITC and Lyso Tracker Red fluorescence (Fig. 4c). These results indicate that RV@Ft-RGD can actively enter cells and then be transferred into the acidic environment of the lysosome (pH ≈ 5.0). These merits, together with the pH-responsive drug release, endow RV@Ft-RGD with many potential applications for in vivo tumor therapy.

### In Vitro Biocompatibility and Tumor Therapy

**Fig. 5:**
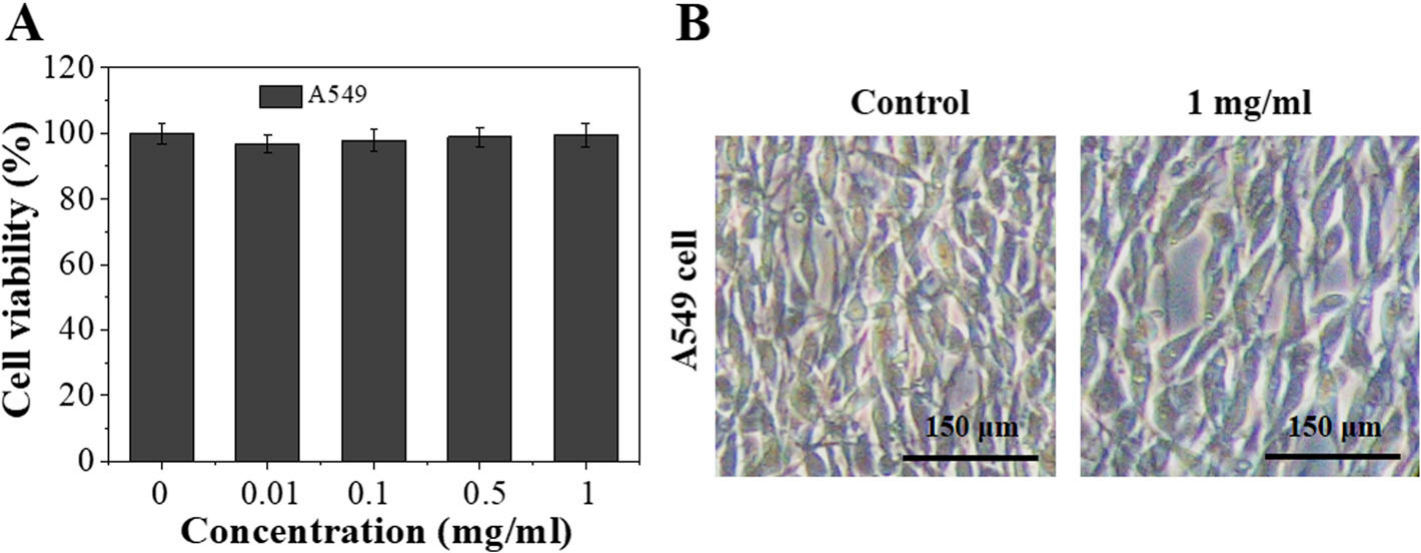
**a** In vitro cytotoxicity against A549 cells treated with different concentration of Ft-RGD for 24 h. **b** The microscope image of A549 cells after 24 h treatment with 1 mg/mL of Ft-RGD

**Fig. 6:**
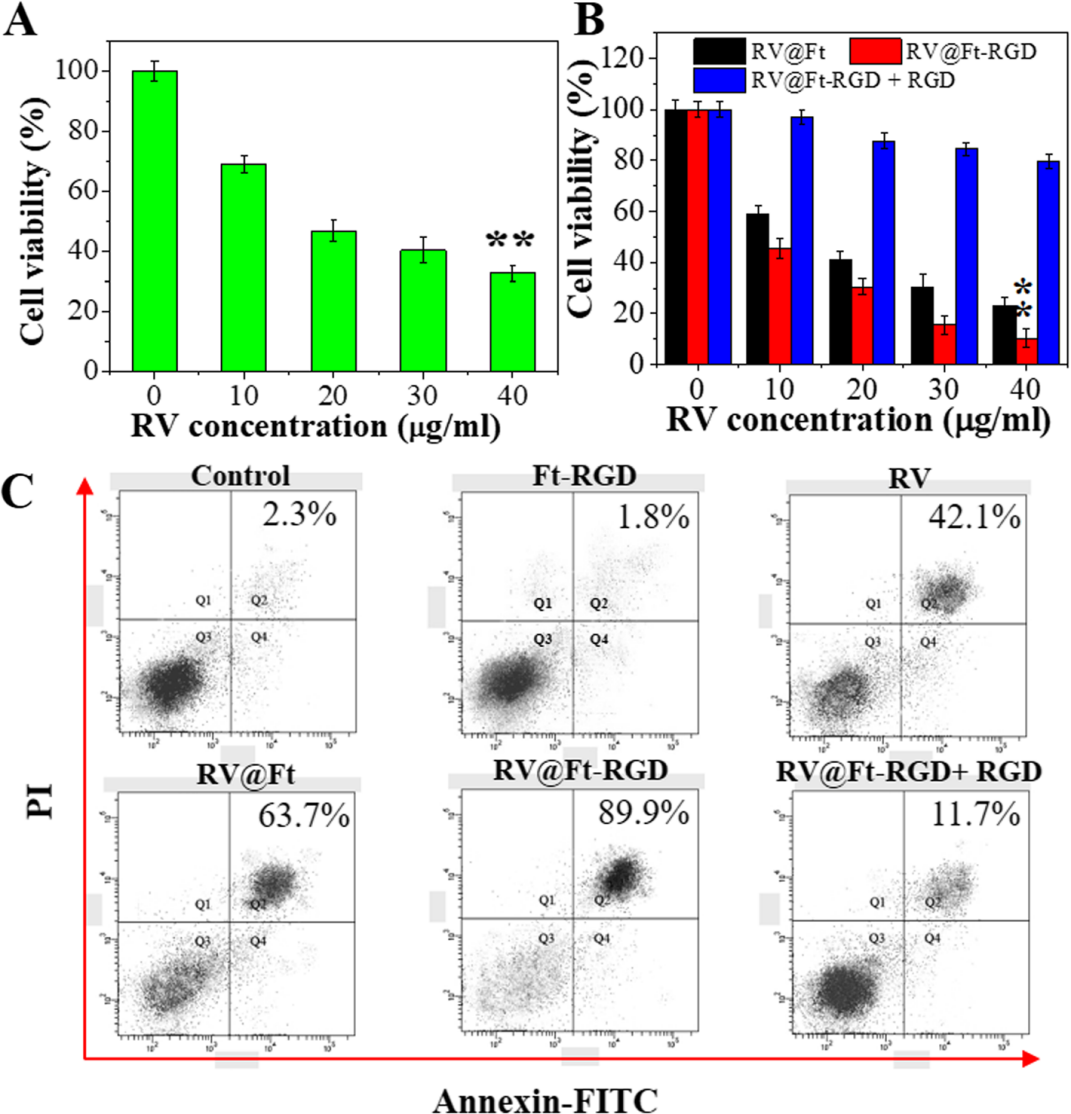
**a** Cell viability of different concentration of RV-treated A549 cells. **b** Cell viability of cells treated with RV@Ft, RV@Ft-RGD + RGD, and RV@Ft-RGD with RV various concentration. **c** Cell apoptosis of A549 cells treated with PBS (control), RV, RV@Ft, RV@Ft-RGD + RGD, and RV@Ft-RGD by flow cytometry. **P < 0.01, compared with other groups, respectively

Prior to studying the antitumor properties of RV@Ft-RGD, the cytotoxicity of the Ft-RGD carrier was investigated. As shown in Fig. 5a and Additional file 1: Figures S2 and S3, Ft-RGD showed no obvious viability suppression to lung cancer A549 cells and NCI-H358 cells at concentrations up to 1 mg/mL. Cell morphology also exhibited no significant change when the 1 mg/mL treated cells were compared to the control groups (Fig. 5b), clearly suggesting that Ft-RGD as a carrier had excellent biocompatibility. As shown in Fig. 6a, b, free RV, RV@Ft, and RV@Ft-RGD all kill the cells in a concentration-dependent manner. RV@Ft-RGD-treated cells showed more viability decrease and only had 11.2% cell viability in 40 μg/mL groups, which was lower than RV@Ft and RV@Ft-RGD + RGD pre-treated groups. A preliminary study with FCM further revealed that with either RV alone, RV@Ft, or RV@Ft-RGD, the majority of the cell deaths was mediated via apoptosis (Fig. 6c). These results demonstrate that the tumor cell-killing effect of RV was greatly enhanced by loading into Ft-RGD, mainly due to increased accumulation of RV@Ft-RGD in the lysosome, where acidic pH condition triggered an increased release of apoptosis-promoting RV into the cytoplasm [32, 33].

### Blood Half-Life of RV@Ft-RGD

**Fig. 7:**
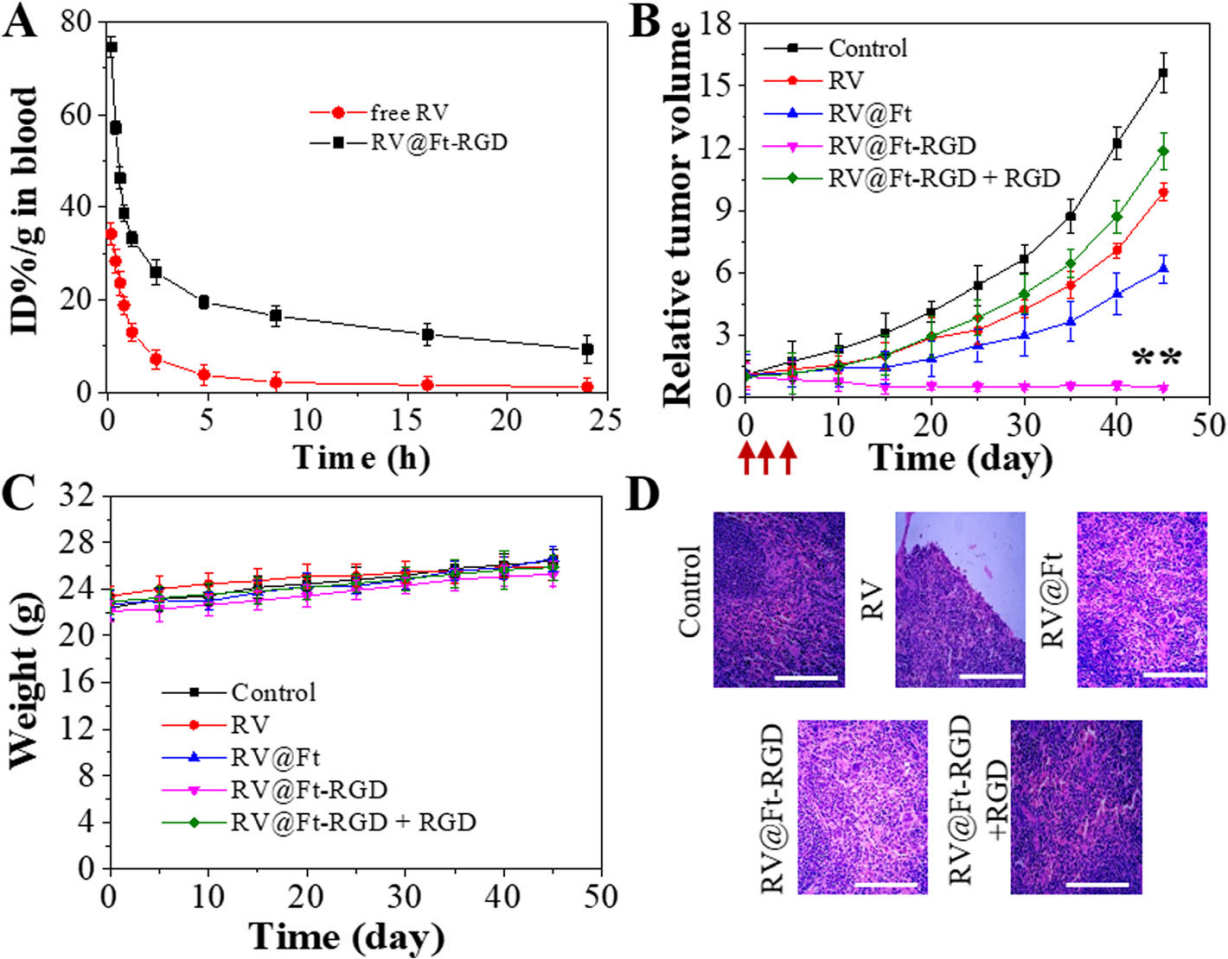
**a** Circulation time of free RV and RV@Ft-RGD in blood. **b** The growth profile of A549 xenografted tumors after three-time intravenous injection of saline (control), RV, RV@Ft, RV@Ft-RGD + RGD, and RV@Ft-RGD. Red arrow indicates the injection time point. **P < 0.01, compared with other groups, respectively. **c** Body weight of tumor-bearing mice after various treatments. **d** Micrographs of H&E-stained tumor slices collected from different groups of mice after the end of the treatment. Scale bars are 50 μm

RV concentration in blood plasma at different times post-injection was studied in free RV and RV@Ft-RGD-treated groups, respectively. As shown in Fig. 7a, the blood half-life of RV@Ft-RGD was 5.1 ± 0.23 h. Free RV had a quicker washout from the circulation, with a blood half-life of only 0.43 ± 0.11 h. The significantly extended half-life of RV@Ft-RGD in the blood is beneficial to improving drug retention in the systemic circulation and facilitates the accumulation of drugs at tumor sites.

### In Vivo Antitumor Effect and Systemic Toxicity of RV@Ft-RGD

**Fig. 8:**
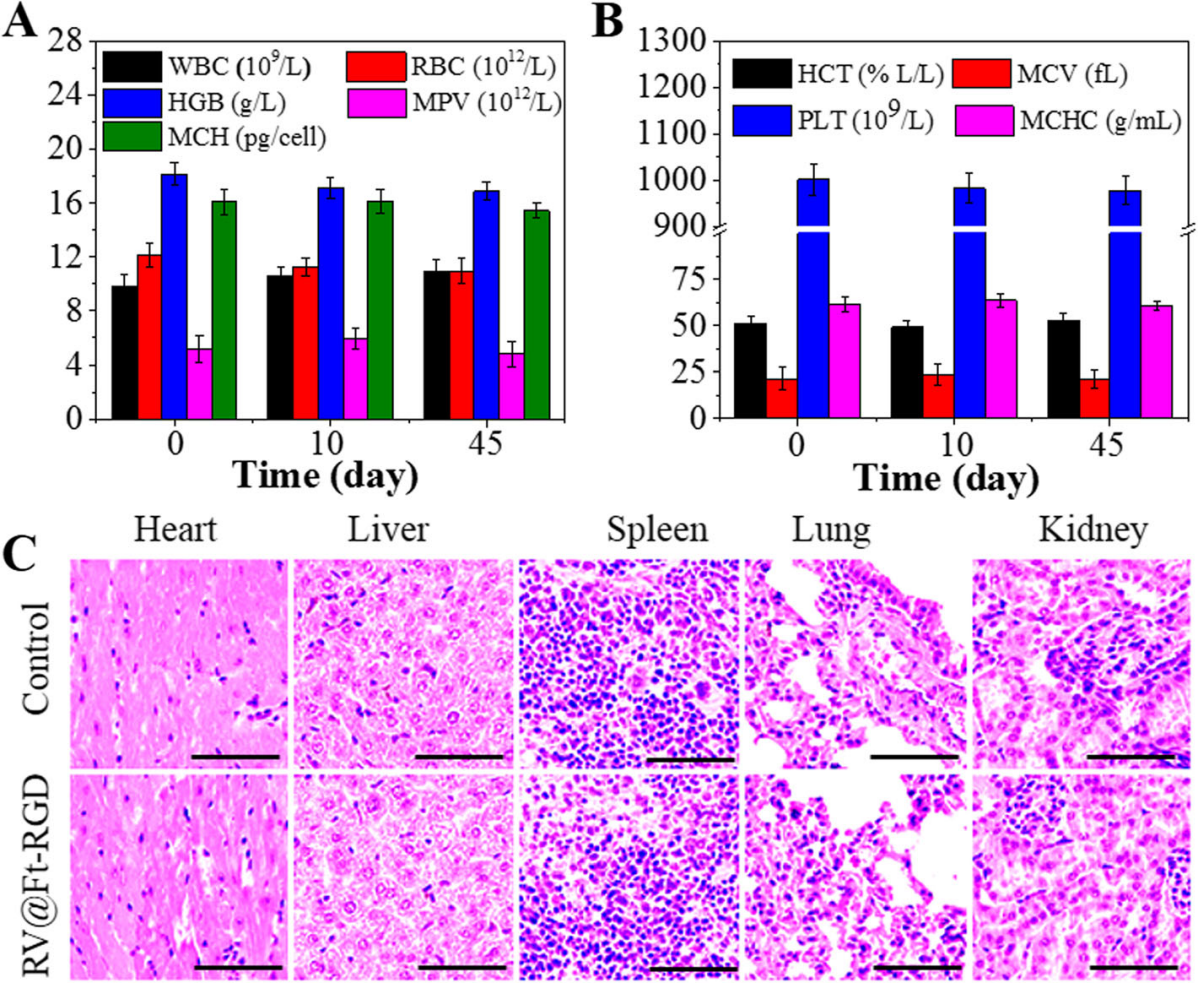
**a** Blood analysis of health mice treated with RV@Ft-RGD for 45 days. **b** Images of H&E-stained major organs of healthy mice treated with RV@Ft-RGD for 45 days. **c** The HE staining images major organs in control and RV@Ft-RGD treated groups. Scale bars are 50 μm

Figure 7b shows the tumor growth profile of tumor-bearing mice with different treatment, which was expressed as relative tumor volume. Tumor growth was inhibited to some degree when treated with RV@Ft, likely due to the low uptake of RV@Ft. However, in the group treated with RV@Ft-RGD, tumor growth was significantly suppressed as compared to the other groups (control, RV, RV@Ft, and RV@Ft-RGD + RGD). During the treatment, there was no noticeable loss of body weight in any experimental group (Fig. 7c), indicating the high bio-safety of RV@Ft-RGD. After the end of the treatment course, H&E staining of tumor tissue in these groups was performed to investigate the chemotherapeutic effects. As shown in Fig. 7d, a large area of apoptosis was observed in tumors treated with RV@Ft-RGD, which is in significant contrast to only a small area of apoptosis in tumors treated with free RV, RV@Ft, and RV@Ft-RGD + RGD. In addition, the systemic toxicity of RV@Ft-RGD was also evaluated in normal mice. Whole blood samples of saline and RV@Ft-RGD-treated health mice were collected at 0, 10, and 45 days post-injection for blood analysis. As shown in Fig. 8a, b, the complete blood counts (WBC, RBC, HGB, MPV, MCH, HCT, MCV, PLT, and MCHC) of RV@Ft-RGD-injected mice showed no significant differences from 0 day to 45 days. The major organs of healthy mice treated with saline and RV@Ft-RGD were also collected at 45 days for H&E staining. No obvious side effects were found in these tissues (Fig. 8c), suggesting negligible long-term adverse toxicity. All of these results demonstrate the promising potential of RV@Ft-RGD for enhanced cancer chemotherapy in vivo.

## Conclusion

In summary, we have successfully developed a pH-induced reversible assembly system (PIRAS) with pH-controllable loading and release of the antitumor RV drug for enhanced tumor-targeting chemotherapy. Under acidic (pH = 5.0) condition, the system can disassemble and load or release RV, while under neutral condition (pH = 7.4), RV@Ft-RGD is highly stable and shows negligible drug leak. Through the RGD-mediated target effect, RV@Ft-RGD can be uptaken in high concentrations by A549 cells and accumulate in the lysosome, which is beneficial for RV release both in vitro and in vivo. Due to the accumulation of the RV@Ft-RGD in the lysosome, and acidic lysosome’s pH-triggering release of RV into the cytoplasm, RV@Ft-RGD showed excellent tumor cell-killing and apoptosis-promoting effects compared to free RV. In addition, after the loading of RV into Ft-RGD, RV@Ft-RGD showed a much longer blood half-time than that of free RV. In vivo results demonstrate that RV@Ft-RGD shows remarkable tumor suppression and no noticeable systemic toxicity. This study suggests that Ft-based PIRAS is highly efficient in drug loading and release for enhanced anticancer therapy.

## Additional files

Additional file 1: **Figure S1. **The TEM image of RV@Ft-RGD. **Figure S2.** In vitro cytotoxicity against NCI-H358 cells treated with different concentration of Ft-RGD for 24 h. **Figure S3.** The microscope image of NCI-H358 cells after 24 h treatment with 1 mg/mL of Ft-RGD. (DOCX 201 kb)

## Data Availability

The conclusions made in this manuscript are based on the data which are all presented and shown in this paper.

## Abbreviations

BSA: Bovine serum albumin
CCK-8: Cell counting kit-8
DLS: Dynamic light scattering
EDC: 1-Ethyl-3-(3-dimethylaminopropyl) carbodiimide
FBS: Fetal bovine serum
FITC: Flourescein isothiocyanate
Ft: Ferritin
HCT: Hematocrit
HGB: Hemoglobin
MCH: Mean corpuscular hemoglobin
MCHC: Mean corpuscular hemoglobin concentration
MCV: Mean corpuscular volume
MPV: Mean platelet volume
NHS: N-hydroxysuccinimide
PIRAS: pH-induced reversible assembly system
PLT: Platelet
RBC: Red blood cells
RGD: Arg-Gly-Asp
RV: Resveratrol
WBC: White blood cell
