# Supplementary material for: A pH-Induced Reversible Assembly System with Resveratrol-Controllable Loading and Release for Enhanced Tumor-Targeting Chemotherapy
Source: Nanoscale Res Lett. 2019 Sep 6;14:305. doi: 10.1186/s11671-019-3139-z (PMC6730982; doi:10.1186/s11671-019-3139-z)
Supplement: Supplementary file 1 — Figure S1. The TEM image of RV@Ft-RGD. Figure S2. In vitro cytotoxicity against NCI-H358 cells treated with different concentration of Ft-RGD for 24 h. Figure S3. The microscope image of NCI-H358 cells after 24 h treatment with 1 mg/mL of Ft-RGD. (DOCX 201 kb) [file 11671_2019_3139_MOESM1_ESM.docx]

**Supplementary Material**

**A pH-induced reversible assembly system with resveratrol-controllable loading and release for enhanced tumor-targeting chemotherapy**

Qingkai Zheng^1^, Wenjing Cheng^2^, Xiaoping Zhang^3^, Runxia Shao^4^, Zhongdong Li^4^*

^1^ Department of respiratory medicine, the people’s hospital of Jiaozuo city, Jiaozuo, Henan 454000, China

^2^ Department of hematopathology, the people’s hospital of Jiaozuo city, Jiaozuo, Henan 454000, China

^3^ Department of physical examination, the people’s hospital of Jiaozuo city, Jiaozuo, Henan 454000, China

^4^ Department of respiratory medicine, the second affiliated hospital of Zhengzhou university, Zhengzhou, Henan 450000, China

*Corresponding author: Zhongdong Li

Email: [lizdzzu_med@hotmail.com](mailto:lizdzzu_med@hotmail.com)

Telephone: + 86-391-2662216


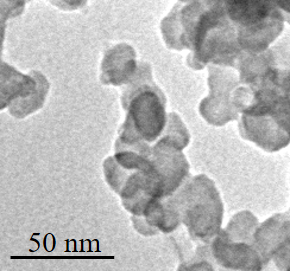


Figure S1 The TEM image of RV@Ft-RGD.


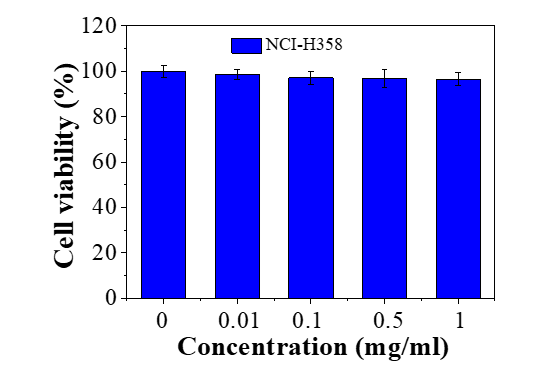


Figure S2 *In vitro* cytotoxicity against NCI-H358 cells treated with different concentration of Ft-RGD for 24 h.


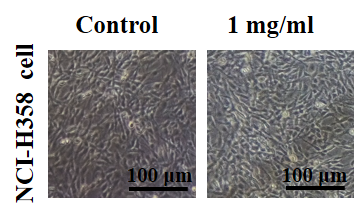


Figure S3 The microscope image of NCI-H358 cells after 24 h treatment with 1 mg/ml of Ft-RGD.
